# *SNTA1*-deficient human cardiomyocytes show shorter field potential duration and slower conduction velocity

**DOI:** 10.1038/s41598-025-16406-6

**Published:** 2025-08-20

**Authors:** Tao Dong, Yan Zhao, Meng Zhang, Wei-Ya Lang, Dan-Yang Liu, Ke-Shuang Zhang, Yue-Jing Wang, Lin Li, Jie Lian, Hong-Bo Yao, Hai-Yan Zhang, Hai-Feng Jin, Tong Lu, Lei Shen, Li-Ling Yue, Yan Lin

**Affiliations:** 1 Department of Anatomy, Histology and Embryology, Basic Medicine School, Qiqihar Medical University, Qiqihar, 161006 Heilongjiang China; 2 Heilongjiang Provincial Key Laboratory of Food & Medicine Homology and Metabolic Disease Prevention, Qiqihar, 161006 Heilongjiang China; 3 Research Institute of Medicine and Pharmacy, Qiqihar Medical University, Qiqihar, 161006 Heilongjiang China; 4 College of Life Science and Agroforestry, Qiqihar University, Qiqihar, 161006 Heilongjiang China; 5 Histology and Embryology Section, Basic Medicine School, Qiqihar Medical University, 333 Bukui Street, Qiqihar, 161006 Heilongjiang China

**Keywords:** Human embryonic stem cell, *SNTA1*-defcient cardiomyocytes, Nav1.5, Field potential duration, Conduction velocity, Embryonic stem cells, Stem-cell differentiation, Arrhythmias, Congenital heart defects

## Abstract

In clinical settings, patients with α-1-syntrophin point mutations are often associated with rare arrhythmias, including Long QT syndrome, Brugada syndrome, and sudden infant death syndrome. Previous studies on α-1-syntrophin have predominantly utilized nonhuman cardiomyocyte models. This study aims to elucidate the phenotype of α-1-syntrophin deficiency using human cardiomyocytes. Using CRISPR/Cas9 technology, we generated *SNTA1* knockout (KO) embryonic stem cell line, which were subsequently differentiated into cardiomyocytes using 2D differentiation method. Genotype analysis identified an adenine (A) insertion in the second exon of *SNTA1*, resulting in a premature stop codon at the 149th amino acid position and truncation within the PDZ domain. *SNTA1*-deficient cardiomyocytes exhibited a shortened field potential duration (FPD) and slower conduction velocity, as detected by micro electrode array analysis. Immunofluorescence analysis further revealed disorganized distribution of Nav1.5 in *SNTA1*-deficient cardiomyocytes. *SNTA1* is a susceptibility locus for arrhythmias and plays a critical role as an essential auxiliary protein in the proper localization of Nav1.5 in human cardiomyocytes.

**Supplementary Information:**

The online version contains supplementary material available at 10.1038/s41598-025-16406-6.

## Introduction

 α−1-syntrophin is a member of the membrane-associated adaptor protein family, consisting of 505 amino acid residues in its unprocessed form, with a molecular weight of approximately 58 kDa^1^. α−1-syntrophin contains three distinct domains: PH1, PH2, and SU. It is predominantly distributed beneath muscle membranes and at neuromuscular junctions^2^. As a peripheral cytoplasmic membrane protein, α−1-syntrophin is primarily associated with dystrophin and dystrophin-related proteins, such as glycoproteins, utrophin, and dystrobrevin, within the dystrophin glycoprotein complex (DGC), also referred to as the dystrophin-associated protein complex (DAPC)^3–5^. Within the DGC, α−1-syntrophin plays a crucial role in supporting the proper subcellular localization of associated functional proteins.

G-proteins are a critical class of signal-transducing proteins located on the inner surface of the cell membrane, where they associate with transmembrane receptors. The N-terminal region of the PH1 domain in α−1-syntrophin and the C-terminal region of the SU domain have been shown to mediate binding with the Gα subunit of heterotrimeric G-proteins, contributing to the regulation of their functions^6^. Calmodulin (CaM) transduces calcium signals by binding to Ca²⁺ ions and interacting with downstream target proteins^7^. The PH1 domain of α−1-syntrophin interacts with CaM, playing a role in intracellular Ca²⁺ regulation^8^. Nav1.5, the primary cardiac voltage-gated sodium channel alpha subunit 5, is essential for the rapid depolarization phase of the cardiac action potential and plays a key role in cardiac conduction. α−1-syntrophin interacts with Nav1.5 through its PDZ domain, which binds to the internal domain of Nav1.5’s N-terminal^9^, as well as through its association with the C-terminal of Nav1.5, thereby contributing to its regulation^10^. Clinically, *SNTA1* has been identified as a susceptibility locus for Long QT Syndrome 12 (LQT12), a rare arrhythmic disorder associated with an increased risk of sudden cardiac death. At present, the etiology and preventive treatment of Long QT Syndrome 12 remain inadequately understood and largely unexplored.

Most current research on α−1-syntrophin relies on animal models; however, significant differences between animal and human cardiac muscle limit their relevance for studying human cardiac arrhythmias. Induced pluripotent stem cell and gene editing technologies offer a powerful platform for creating precise human disease models to advance precision medicine^11^. Therefore, we utilized CRISPR/Cas9 technology to generate *SNTA1* knockout (SNTA1-KO) human cardiomyocytes, providing a more physiologically relevant model for investigating arrhythmias resulting from α−1-syntrophin deficiency. Our present work establishes a foundation for studying α−1-syntrophin point mutations.

## Results

### Establishment of homozygous ***SNTA1***-deficient human embryonic stem cell

**Fig. 1:**
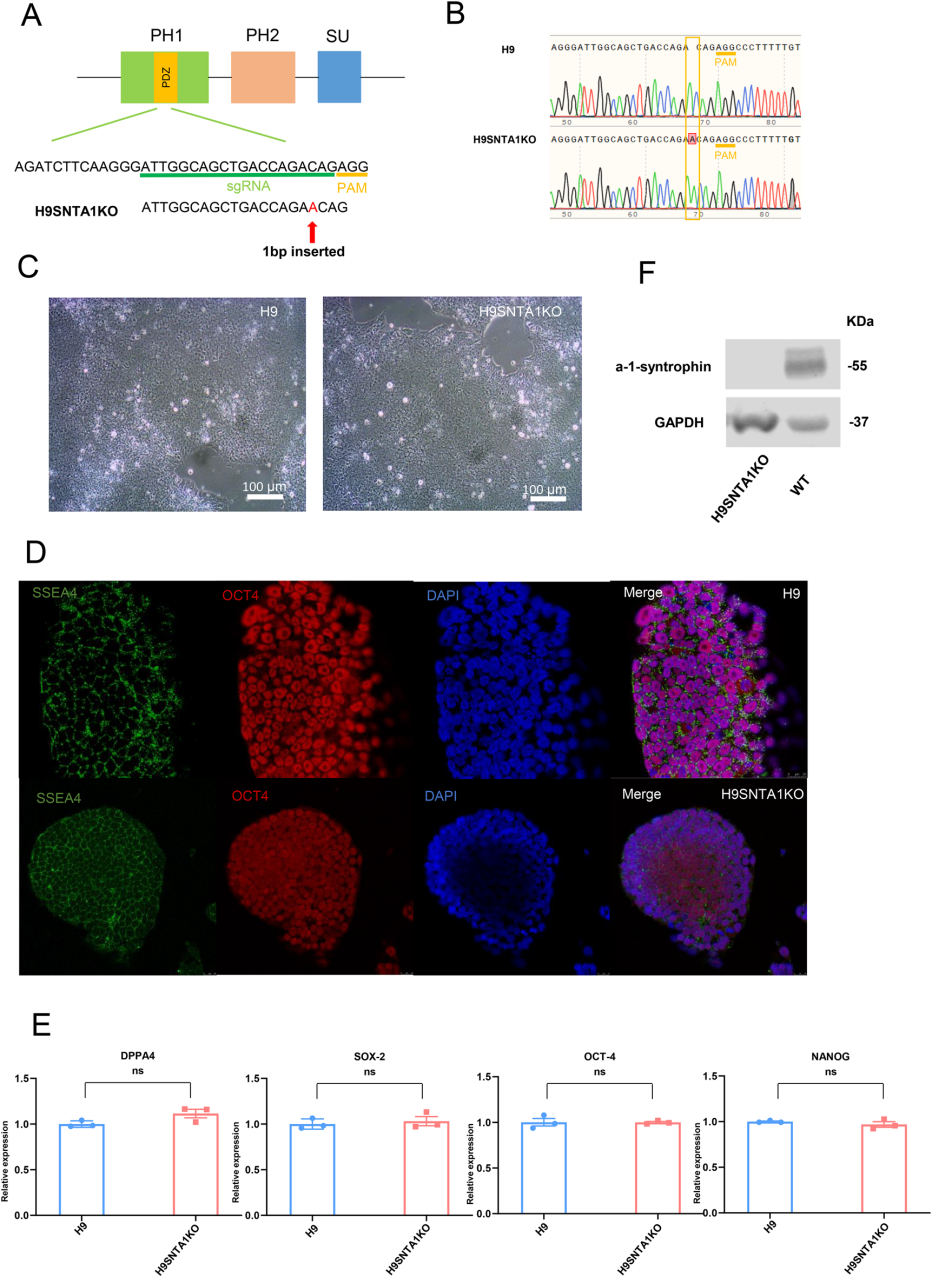
Establishment of homozygous *SNTA1*-deficient human embryonic stem cell. A. Schematic of the sgRNA designed to the PH1 domain in *SNTA1*.There is one adenine nucleotide inserted into *SNTA1* before PAM sequence. B. The sanger sequence of H9 and H9SNTA1KO genomic DNA. There is one adenine nucleotide inserted into the second exon of *SNTA1* in H9SNTA1KO. C. The light microscope images of H9 and H9SNTA1KO. Scale bar: 100 μm. D. Immunofluorescence staining for pluripotency was performed. Both SSEA4 and OCT-4 were positive in H9 and H9SNTA1KO. *SNTA1*-knockout did not influence the pluripotency of hESCs. Scale bar: 25 μm. E. The stem cell pluripotency qPCR results of DPPA-4, SOX-2, OCT-4, and NANOG. There is no significance between H9 and H9SNTA1KO. (*N* = 3). ns; not significant, unpaired two-sided Student’s t test. F. Western blot analysis indicates that cardiomyocytes derived from H9SNTA1KO cells are deficient in α−1-syntrophin expression.

We selected the second exon, a shared exon, as the target for *SNTA1* editing. A single guide RNA (sgRNA) was designed to target this exon, with the sequence 5′-ATTGGCAGCTGACCAGACAG-3′, and the protospacer adjacent motif (PAM) sequence AGG (Fig. 1A). The sgRNA was ligated into a linear CRISPR/Cas9 plasmid, followed by plasmid amplification. The resulting plasmid was delivered into H9 embryonic stem cells via electroporation using the LONZA Nucleofector 4D system. Post-electroporation, the cells were selected using 0.3 µg/mL puromycin. Resistant clones were picked, expanded, and subjected to genomic DNA extraction for genotyping analysis. One clone was identified with an adenine nucleotide insertion upstream of the PAM region (Fig. 1B). This insertion introduced a premature stop codon (TGA) at amino acid position 149, truncating the α−1-syntrophin protein in the PDZ domain which is located in the PH1 domain. This modified cell line was designated as H9SNTA1KO^12^. The H9SNTA1KO cells were cultured and compared to the parental H9 embryonic stem cells under light microscopy, revealing no discernible morphological differences (Fig. 1C). Immunofluorescence staining confirmed positive expression of the pluripotency markers SSEA4 and OCT4 in H9 and H9SNTA1KO cells (Fig. 1D). Furthermore, qRT-PCR analysis demonstrated that the expression levels of the pluripotency markers DPPA4, SOX2, OCT4, and NANOG in H9SNTA1KO cells were comparable to those in H9 cells, with no significant differences observed (Fig. 1E). Western blot analysis further confirmed that cardiomyocytes derived from H9SNTA1KO cells exhibit a deficiency in α−1-syntrophin (Fig. 1F and Supplementary Material 1 Figure.1 A). All results confirmed the successful establishment of H9SNTA1KO cells, which retained normal pluripotency markers and *SNTA1* deficiency.

#### H9SNTA1KO differentiation to cardiomyocytes

**Fig. 2:**
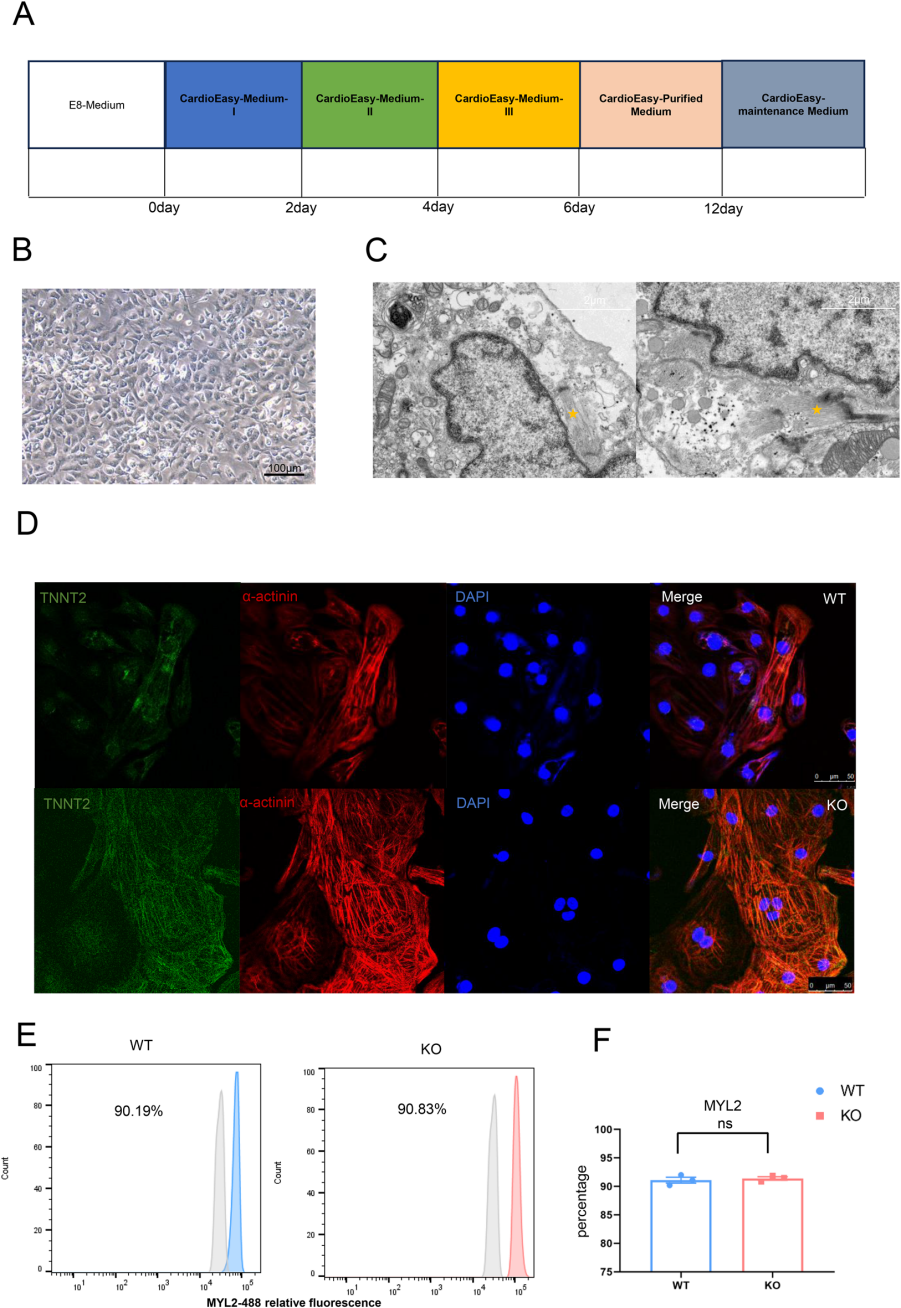
H9SNTA1KO differentiation to cardiomyocytes. A. Schematic of embryonic stem cell induction into cardiomyocytes using small molecular inhibitors two-dimensional differentiation method. B. The light microscope image of beating KO cardiomyocytes at day 10 of differentiation. Scale bar: 100 μm. C. The transmission electron microscope image of KO cardiomyocyte at 30days. The yellow pentagram shows the myofilaments in cardiomyocyte. Scale bar: 2 μm. D. Immunofluorescence staining of TNNT2 (green) and α-actinin (red) in WT and KO cardiomyocytes. Scale bar: 50 μm. E. Flow cytometry was used to detect a specific ventricular muscle marker, MYL2. The results demonstrated that the yield of WT and KO ventricular muscle was similar when purified by metabolic selection. F. Quantification of MYL2 of the flow cytometry (*N* = 3). ns; not significant, unpaired two-sided Student’s t test.

To obtain cardiomyocytes, we employed a 2D differentiation method as described in previous studies^13,14^. The process of differentiation required approximately 12 days (Fig. 2A). Cardiomyocytes were collected at day 30 of differentiation (Fig. 2B). Transmission electron microscopy (TEM) analysis revealed the presence of myofibrils in the cytoplasm (Fig. 2C, yellow pentagrams), although the cells were immature, lacking T-tubules and well-organized layered myofilaments. Immunofluorescence staining was performed to detect the cardiomyocyte-specific markers TNNT2 and α-actinin, both of which were positively expressed in H9-derived cardiomyocytes (WT cardiomyocytes) and H9SNTA1KO-derived cardiomyocytes (KO cardiomyocytes) (Fig. 2D). The collected cells were identified as cardiomyocytes, which exhibited spontaneous contractions. The ventricular muscle-specific marker MYL2 was assessed in both WT cardiomyocytes and KO cardiomyocytes after purified medium selection using flow cytometry (Fig. 2E). The results demonstrated that H9SNTA1KO cells differentiated into cardiomyocyte subtypes comparable to WT cells, confirming normal differentiation capacity (Fig. 2F). All results confirmed the successful generation of H9SNTA1KO cardiomyocytes, which closely resemble those of WT.

## H9SNTA1KO derived cardiomyocytes showed shorter field potential duration and slower conduction velocity

**Fig. 3:**
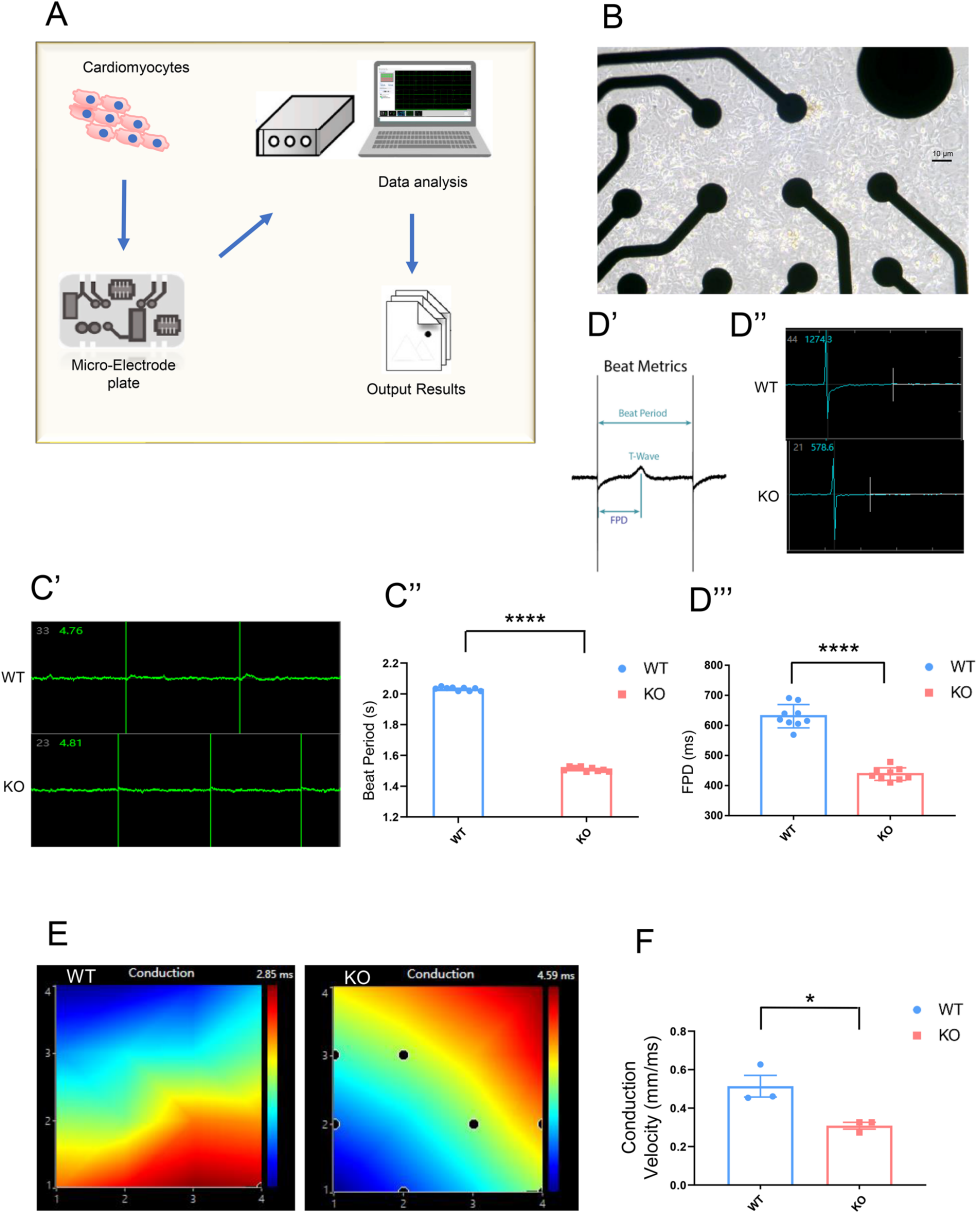
H9SNTA1KO derived cardiomyocytes showed shorter field potential duration and slower conduction velocity. **A**. Schematic of MAESTRO™ MEA SYSTEMS work flow. **B**. The image of cardiomyocytes seeded on the Micro-electrode plate. Scale bar: 10 μm. C’. The image of Continuous Waveform Plots for WT cardiomyocytes and KO cardiomyocytes. C’’. The statistical of beat period of the WT cardiomyocytes and KO cardiomyocytes. There is a statistical difference between them. *N* = 9, ****P < 0.0001. The beat period of KO cardiomyocytes was shorter than that of WT. D’. The Schematic of beat Metrics. D’’. The image of cardiac voltage waveforms for WT cardiomyocytes and KO cardiomyocytes. D’’’. The statistical FPD diagram of WT cardiomyocytes and KO cardiomyocytes. The MEA results indicates that the FPD of KO cardiomyocytes is shorter than that of WT. There is a statistical difference. *N* = 9, ****P < 0.0001. E. The propagation map of WT cardiomyocytes and KO cardiomyocytes. F. The MEA results showing the conduction velocity of KO cardiomyocytes is slower than the WT cardiomyocytes. There is a statistical difference. *N* = 3, *P < 0.05.

*SNTA1*, known as the LQT12 gene, has been associated with long QT syndrome (LQTS)^15,16^. LQTS, a hereditary condition characterized by QT interval prolongation, increases the risk of life-threatening arrhythmias. To investigate the effects of α−1-syntrophin deficiency in cardiomyocytes, we employed the MAESTRO™ MEA SYSTEMS to assess their electrical activity (Fig. 3A). At day 30 of differentiation, cardiomyocytes were seeded onto MEA (micro electrode array) plates for electrical activity analysis under normal culture conditions (Fig. 3B). Data from the MAESTRO™ MEA SYSTEMS demonstrated that the beat period of KO cardiomyocytes (1.510 ± 0.005 s) was significantly shorter than that of WT cardiomyocytes (2.028 ± 0.004 s). (Fig. 3C’-C’’). The interval between depolarization and repolarization, referred to as the field potential duration (FPD), was extracted from field potential signals (Fig. 3D’-D’’). “Statistical analysis demonstrated a significant reduction in FPD of KO cardiomyocytes (438.13 ± 6.98 ms) compared to that of WT cardiomyocytes (630.49 ± 12.91 ms). (Fig. 3D’’’). The results showed there are abnormal FPD in the KO cardiomyocytes. The stability of beat propagation patterns and conduction velocity was also assessed to evaluate the functional properties of the cardiomyocytes (Fig. 3E). Statistical analysis demonstrated that the conduction velocity of KO cardiomyocytes (0.309 ± 0.018 mm/ms) was significantly slower than that of WT cardiomyocytes (0.514 ± 0.057 mm/ms) (Fig. 3F). Reduced conduction velocity may contribute to an increased risk of arrhythmias^16^. All the results showed there is a potential risk of arrhythmia in KO cardiomyocytes.

## H9SNTA1KO derived cardiomyocytes showed the disorganization of Nav1.5

**Fig. 4:**
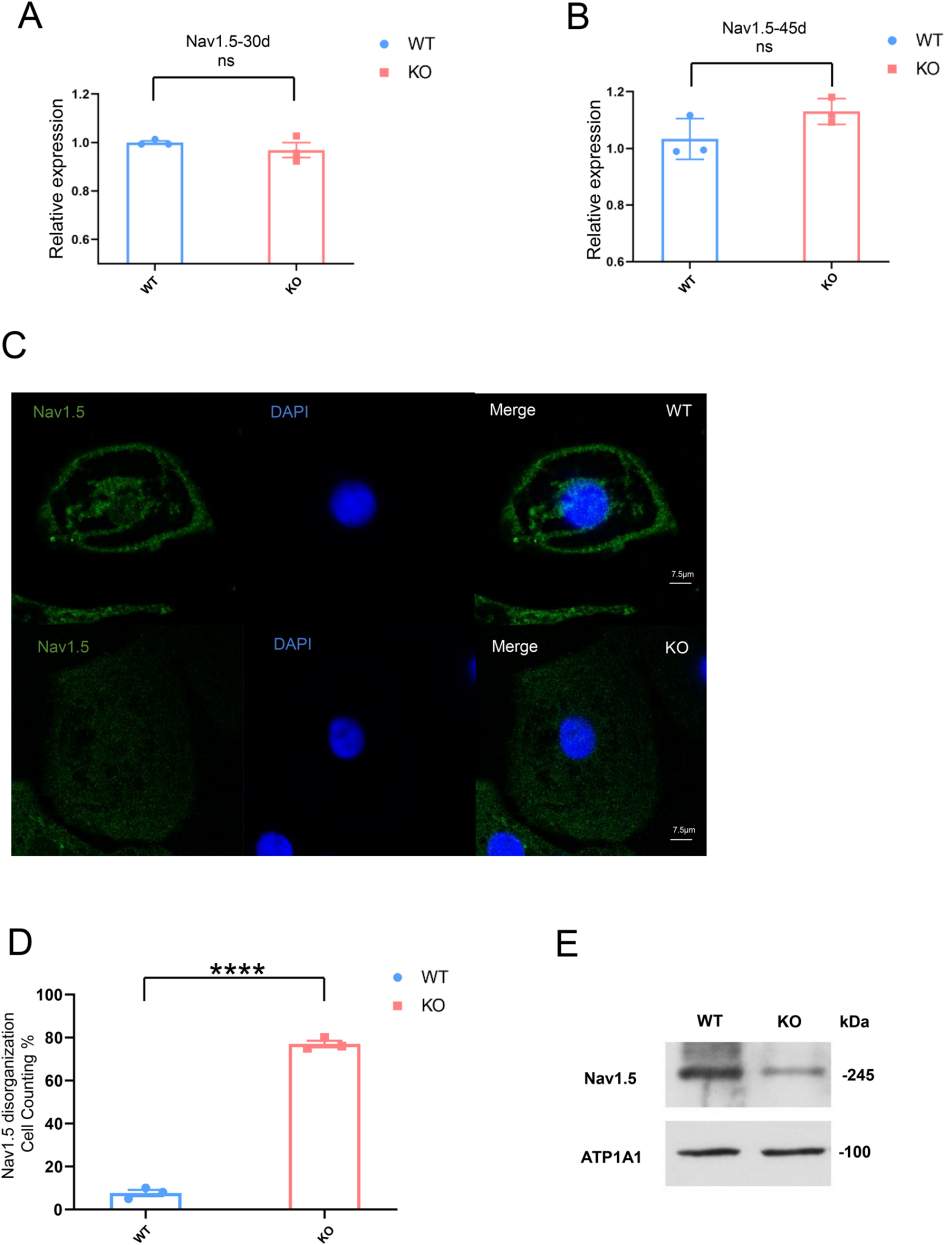
H9SNTA1KO derived cardiomyocytes showed the disorganization of Nav1.5. A. The expression of Nav1.5 in 30-day WT and KO cardiomyocytes was analyzed using RT-qPCR. The results showed there is no significant. ns; not significant. B. The expression of Nav1.5 in 45-day WT and KO cardiomyocytes was analyzed using RT-qPCR. The results showed there is no significant. ns; not significant. C. Immunostaining of Nav1.5 in 45-day WT and KO cardiomyocytes. Scale bar: 7 μm. D. Statistical analysis of Nav1.5 disorganization cell counting in 45-day WT and KO cardiomyocytes. The results showed that KO cardiomyocytes exhibit a higher number of disorganized cells compared to WT cardiomyocytes. ****P < 0.0001. E. Western blotting of Nav1.5 on cell membrane of WT and KO cardiomyocytes at day 45 of differentiation.

α−1-syntrophin interacts with both the C-terminal and N-terminal domains of Nav1.5^9^, facilitating the proper membrane localization of Nav1.5^10^. To investigate this further, we conducted additional experiments. mRNA was extracted from 30-day-old cardiomyocytes, and Nav1.5 expression was analyzed. The results indicated no significant difference in Nav1.5 expression between the WT and KO cardiomyocytes at 30 days (Fig. 4A). Similarly, mRNA extracted from 45-day-old cardiomyocytes showed no difference in Nav1.5 expression between the WT cardiomyocytes and KO cardiomyocytes (Fig. 4B). These findings suggested that α−1-syntrophin deficiency does not influence Nav1.5 transcription. The cellular localization of Nav1.5 was further examined using immunofluorescence staining (Fig. 4C). The imaging results revealed a disorganized distribution of Nav1.5 in KO cardiomyocytes compared to the WT cardiomyocytes. Statistical analysis confirmed that the proportion of cells with disorganized Nav1.5 localization was significantly higher in the KO cardiomyocytes than in the WT cardiomyocytes (Fig. 4D). We analyzed Nav1.5 expression on the cell membrane of WT and KO cardiomyocytes using Western blotting (Fig. 4E and Supplementary Material 1 Figure.1B). The results demonstrated a reduction in Nav1.5 expression on the cell membrane in KO cardiomyocytes compared to WT cardiomyocytes.

## Discussion

As an adapter protein, α−1-syntrophin is localized beneath the cell membrane and contains three functional domains: PH1, PH2, and SU. These domains are involved in the subcellular localization of various intracellular functional proteins, including the Gα subunit, CaM, and Nav1.5^7–10^. Clinical reports have indicated that mutations in α−1-syntrophin may be associated with Long QT Syndrome (LQTS)^15^ and other cardiovascular phenotypes. Long QT syndrome (LQTS) encompasses a group of heritable conditions characterized by cardiac repolarization dysfunction^16^. To investigate the effects of α−1-syntrophin mutations, we employed a human cell model. Using gene editing system, we targeted the second exon of *SNTA1* in H9 embryonic stem cells. Following gene editing, we established a *SNTA1*-deficient human embryonic stem cell line. Genotyping results revealed an adenine nucleotide insertion in the second exon of *SNTA1*, resulting in a premature termination of the protein. Thus, the H9SNTA1KO embryonic stem cell line was successfully generated^12^. The morphology of the H9SNTA1KO cell line was similar to that of the parental H9 cells, and both cell lines exhibited normal expression of pluripotency markers. We then used a chemically defined 2D differentiation method to induce cardiomyocyte differentiation^13,14^. No significant differences were observed between H9SNTA1KO-derived cardiomyocytes and those derived from H9 embryonic stem cells in terms of differentiation efficiency.

To assess the electrical activity of cardiomyocytes, we employed the MAESTRO™ MEA SYSTEMS. The cardiomyocytes were seeded onto the MEA plate, and the data showed that the KO cardiomyocytes exhibited a shorter beat period compared to the WT cardiomyocytes. Additionally, the KO cardiomyocytes had a shorter field potential duration (FPD) and slower conduction velocity. FPD refers to the duration of the field potential recorded using a Microelectrode Array (MEA) in multicellular culture systems, such as hESCs-derived cardiomyocytes. It reflects the electrophysiological activity of two-dimensional tissue, analogous to the QT interval observed in a surface electrocardiogram (ECG). The shorter FPD observed in the KO cardiomyocytes indicates an abnormality in the depolarization and repolarization process. Clinically, alterations in the QT interval can lead to arrhythmias^17^. Furthermore, the KO cardiomyocytes exhibited a slower conduction velocity. The measured conduction velocity reflects the collective effects of various factors, including cell culture health and pacemaker stability. Slower conduction propagation can potentially contribute to arrhythmias^18^. These results provide evidence that the electrical activity of *SNTA1*-deficient cardiomyocytes is unstable, potentially increasing the risk of cardiac arrhythmias.

To further explore the underlying cause of the shorter FPD and slower conduction propagation in *SNTA1*-deficient cardiomyocytes, we focused on Nav1.5. Nav1.5 is a voltage-gated sodium ion channel alpha subunit 5 located on the cardiac membrane, playing a crucial role in the depolarization process of cardiomyocyte action potentials^19^. While the expression of Nav1.5 showed no significant difference between KO and WT cardiomyocytes, α−1-syntrophin acts as an adaptor protein that helps target functional proteins to the cell membrane. To investigate the localization of Nav1.5 in cardiomyocytes, immunofluorescence staining was performed. The results revealed that Nav1.5 localization was more disorganized in KO cardiomyocytes compared to WT cardiomyocytes. These findings suggest that the proper localization of Nav1.5 in cardiomyocytes requires α−1-syntrophin. Based on these observations, we speculate that α−1-syntrophin deficiency may impair Nav1.5 localization, potentially increasing the likelihood of arrhythmia in the KO cardiomyocytes. However, the specific mechanisms underlying this effect require further investigation.

## Limitation

This study evaluated the electrical activity of cardiomyocytes at the population level using multi-electrode array recordings. However, single-cell electrophysiological measurements were not conducted, and patch-clamp validation of ion channel function was not included. Additionally, the cardiomyocytes used in this study appeared to lack well-developed T-tubule structures, suggesting a relatively immature phenotype.

## Conclusion

In this study, a human *SNTA1*-knockout cell model was established using the CRISPR/Cas9 system. This cell model provides a valuable tool for studying arrhythmias induced by α−1-syntrophin deficiency in vitro. The findings underscore the critical role of α−1-syntrophin as an essential auxiliary protein involved in the proper localization of Nav1.5, a key cardiac ion channel in cardiomyocytes. *SNTA1* is identified as a susceptibility locus for arrhythmias, highlighting its potential as a target for further research in cardiac electrophysiology.

## Methods

### Embryonic stem cell culture

H9 embryonic stem cells (Product Information of the cell line in Supplementary Material 2) (a gift from Feng Lan Professor) were cultured in E8 medium and passaged using 0.5 mM EDTA upon reaching 80% confluence. Cells were typically passaged at a ratio of 1:6.

### Establishment the SNTA1KO embryonic stem cell line

 Using the Zhang Lab’s resources, we designed an sgRNA targeting the second exon of *SNTA1*. The sgRNA sequence was 5′- ATTGGCAGCTGACCAGACAG − 3′. This sequence was ligated into a CRISPR/Cas9 plasmid, which was subsequently amplified by transforming E. coli cells (Top10 Competent Cells, CWBIO, China) and purified using an EndoFree Mini Plasmid Kit (TianGen, China). The plasmid was then delivered into H9 embryonic stem cells via electroporation (LONZA Nucleofector 4D). Following puromycin selection, individual clones were isolated and subjected to genotypic identification. An adenine insertion was introduced into the second exon, resulting in a premature stop codon at the 149th amino acid position of α−1-syntrophin. Primer Premier 6.0 and SnapGene 2.3.2 were used to carry out this process (The software URL was provided in Table S3). This successfully established the H9SNTA1KO cell line^12^.

### Cardiac differentiation

Embryonic stem cells (ESCs) were cultured in E8 medium. When the cells reached approximately 70–80% confluence, they were passaged at a 1:6 ratio using E8 medium supplemented with 10 µM Y-27,632 (ROCK inhibitor, MCE, USA). Once the cells reached 80–90% confluence, cardiac differentiation was used 2D differentiation method. The CardioEasy^®^ mediums for differentiation were provided by the Cellapybio Inc (China).

### Flow cytometry

Cardiomyocytes were digested using CardioEasy^®^ I and CardioEasy^®^ II digestive solutions (Cellapybio, China) to prepare single-cell suspensions. The cells were then fixed in 4% paraformaldehyde for 15 min at room temperature (RT), followed by two washes with PBS. Permeabilization was performed using 0.2% Triton X-100 for 5 min at RT. Next, the cells were incubated with the antibody for 30 min in the dark at RT, followed by two additional PBS washes to remove unbound antibodies. The samples were analyzed using a flow cytometer (Beckman, EPICS XL), and the results were processed with FlowJo_V10.8.1 (The software URL was provided in Table S3).

### Immunofluorescent staining

 Immunofluorescence staining was performed to visualize the localization of intracellular antigens. Cells were seeded on coverslips and cultured until reaching approximately 50% confluence. The medium was aspirated, and the cells were washed three times with PBS. The coverslip-adherent cells were fixed in 4% paraformaldehyde for 30 min at room temperature (RT), followed by three washes with PBS. Permeabilization was performed using 0.3% Triton X-100 for 10 min at RT. The cells were then blocked with 3% BSA for 30 min at RT. After blocking, the cells were incubated with the primary antibody at 4 °C for 24 h. Following three PBS washes, they were incubated with the secondary antibody and DAPI (100 nM) for 1 h at RT. The cells were subsequently washed three more times with PBS and imaged using a confocal microscope (Leica, TCS SP5). Both primary and secondary antibodies were used for immunofluorescence staining. Both primary and secondary antibodies were provided in Table S2. The results were processed with ImageJ_v1.8.0 (The software URL was provided in Table S3).

### Quantitative real‑time PCR (qRT‑PCR)

 To compare gene expression at the transcriptional level between the KO and WT groups, real-time PCR was performed. Cells were seeded in a 6-well plate at a density sufficient to reach approximately 90% confluence for RNA extraction. Total RNA was extracted using TRIzol reagent (Invitrogen, USA) and treated with DNase I (Beyotime, China) at 37 °C for 30 min to remove any contaminating DNA. Reverse transcription was performed using the PrimeScript™ reverse transcription system (TaKaRa, Japan). Relative gene expression levels were analyzed by quantitative real-time PCR (qRT-PCR) on an iCycler iQ5 system (Bio-Rad, USA) using TB Green™ Premix Ex Taq™ II (TaKaRa, Japan). Relative quantification of gene expression was determined using the ∆CT method. Primer sequences used for qRT-PCR are provided in Table S1.

### Electrical activity of cardiomyocytes detection

To assess the electrical activity of cardiomyocytes under normal culture conditions, we utilized the MAESTRO™ MEA SYSTEMS. Matrigel working solution was prepared by diluting it 1:200 and used to coat the wells of a 24-well MEA plate overnight. A total of 20,000–30,000 cardiomyocytes were seeded onto one matrigel-coated well of the 24-well MEA plate and cultured in cardiac maintenance medium (Cellapybio, China) supplemented with 10 µM Y-27,632. Once the cardiomyocytes spread and began beating regularly, their electrical activity was measured and analyzed using the MAESTRO™ MEA SYSTEMS(Axion BioSystems, Inc, US).

### Transmission electron microscope (TEM)

Transmission electron microscopy was employed to examine the ultrastructure of cardiomyocytes. The medium was aspirated from the cardiomyocytes, and without rinsing, they were immediately fixed in 2.5% glutaral solution. The cells were then gently scraped off using a cell scraper and collected into a centrifuge tube (cells avoid being digested by enzymes). After centrifugation, a visible cell pellet should be obtained. The cells were fixed at RT for 2 h with fresh electron microscope fixative. The samples were subsequently sent to Wuhan GoodBio Technology Company for further processing.

### Western blotting

The protein was extracted using the Membrane Protein Extraction Kit (Cat. No. PK10015, Proteintech, USA). Cells are rinsed in cold PBS three times, and carefully remove the residual PBS as much as possible. Then, on ice, directly add 500uL of cold Membrane Protein Extraction Reagent A for about 4 million cells. Vortex the samples containing Reagent A vigorously on a vortex mixer for 1 min (10 s on, 10 s off), then incubate the samples on ice for 2 min. Repeat the vortexing and incubation steps four times in total. Centrifuge the homogenized samples at 700×g for 10 min at 4 °C. Carefully collect the supernatant (avoid disturbing the pellet). Centrifuge the collected supernatant at 16,000×g for 30 min at 4 °C. The pellet contains the cell membrane fraction, and the supernatant contains cytosolic proteins. Carefully Collect the cell membrane fraction. Resuspend the cell membrane fraction pellet in 50 µL of Membrane Protein Extraction Reagent B. Vortex vigorously at high speed (10 s on, 10 s off) for a total of 2 min, then incubate on ice for 10 min. Repeat the vortexing and ice incubation four times to ensure thorough extraction of membrane proteins. Centrifuge the samples at 16,000×g for 10 min at 4 °C, and collect the supernatant, which contains the cell membrane protein. The SDS-PAGE protein-loading buffer (Beyotime, China) was added, and the protein were heat-denatured in a 37 °C water bath for 45 min. Based on the molecular weight of the target protein, we performed SDS-PAGE gel electrophoresis, followed by protein transfer onto a polyvinylidene difluoride (PVDF) membrane using a Bio-Rad gel transfer system. Then, we blocked the membrane with 5% skimmed milk for 1 h at room temperature. The membrane was incubated with the primary antibody overnight at 4 °C, followed by incubation with the secondary antibody for 2 h at room temperature. Both primary and secondary antibodies were provided in Table S2.

### Statistical methods

The data are presented as mean ± standard deviation. Differences between two groups were analyzed using a one-tailed or two-tailed t-test, while rates were compared using Fisher’s Exact test. For comparisons involving three or more groups, one-way or two-way analysis of variance (ANOVA) was applied, followed by Tukey’s multiple comparison test. A 95% confidence interval was used, and statistical significance was defined as follows: *P < 0.05, **P < 0.01, ***P < 0.001, and ****P < 0.0001, representing four levels of significance.

All statistical analyses were conducted using GraphPad Prism 8.0 (The software URL was provided in Table S3).

## Supplementary Information

Below is the link to the electronic supplementary material.

Supplementary Material 1

Supplementary Material 2

Supplementary Material 3

## Data Availability

The datasets used and/or analyzed during the current study available from the corresponding author on reasonable request.

## Abbreviations

SNTA1: The gene of syntrophin alpha 1
$\alpha$-1-syntrophin: The protein of syntrophin alpha 1
hESCs: human embryonic stem cells
H9SNTA1KO: SNTA1-deficient H9 embryonic stem cells
WT: H9 embryonic stem cells
WT cardiomyocytes: cardiomyocytes derived from H9 embryonic stem cells
KO: SNTA1-deficient H9 embryonic stem cells or H9SNTA1KO
KO cardiomyocytes: SNTA1-deficient cardiomyocytes
PAM: protospacer adjacent motif
CRISPR/Cas9: clustered regularly interspaced short palindromic repeats/CRISPR-associated protein 9
TNNT2: troponin T2, cardiac type
SSEA4: stage-specific embryonic antigen-4
NANOG: Nanog homeobox
SOX2: SRY-box transcription factor 2
DPPA4: developmental pluripotency associated 4
OCT-4: POU class 5 homeobox 1
MYL2: myosin light chain 2
MEA: micro electrode array
SCN5A: The gene of sodium voltage-gated channel alpha subunit 5
Nav1.5: The protein of sodium voltage-gated channel alpha subunit 5
