# Supplementary material for: SNTA1-deficient human cardiomyocytes show shorter field potential duration and slower conduction velocity
Source: Sci Rep. 2025 Aug 20;15:30600. doi: 10.1038/s41598-025-16406-6 (PMC12367991; doi:10.1038/s41598-025-16406-6)
Supplement: Supplementary file 1 — Supplementary Material 1 [file 41598_2025_16406_MOESM1_ESM.docx]

Supplementary Figure.1A


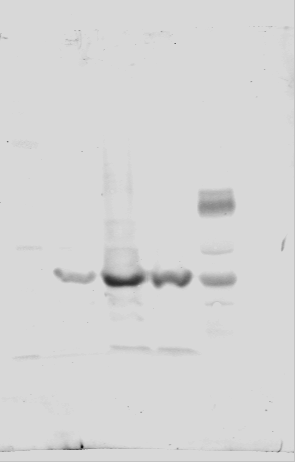


Supplementary Figure.1A is the original, unprocessed version of Figure 1F.

Supplementary Figure.1B


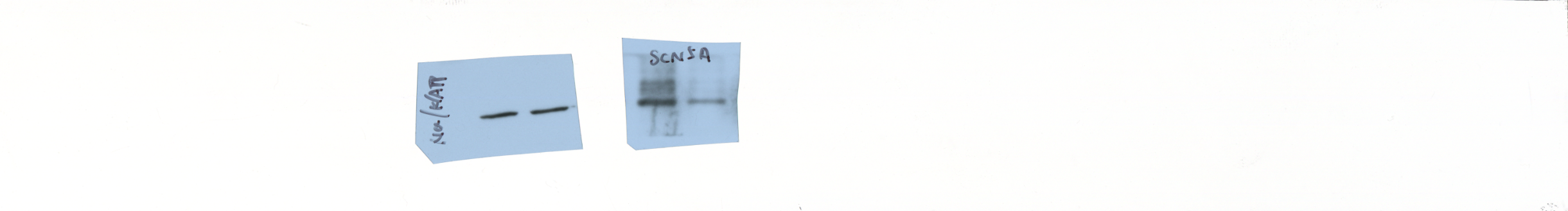


Supplementary Figure.1B is the original, unprocessed version of Figure 4E.
