# Supplementary material for: SNTA1-deficient human cardiomyocytes show shorter field potential duration and slower conduction velocity
Source: Sci Rep. 2025 Aug 20;15:30600. doi: 10.1038/s41598-025-16406-6 (PMC12367991; doi:10.1038/s41598-025-16406-6)
Supplement: Supplementary file 3 — Supplementary Material 3 [file 41598_2025_16406_MOESM3_ESM.docx]

Table S1. Primer sequences used for q-PCR

| Gene | Forward 5’-3’ | Reverse 5’-3’ |
| --- | --- | --- |
| DPPA4 | GCAAAAGGCAAGGAGGAACATC | CTGGGGTTGTCAGTGTGCTC |
| SOX-2 | AACCAGCGCATGGACAGTTA | CGAGCTGGTCATGGAGTTGT |
| OCT-4 | CTCGAGAAGGATGTGGTCCG | AGCCTGGGGTACCAAAATGG |
| NANOG | AATGGTGTGACGCAGGGATG | TGCACCAGGTCTGAGTGTTC |
| SCN5A | CTGACCTCACCATCACTATGTG | GCTGTGAAAATCCCTGTGAAG |
| GAPDH | GGAGCGAGATCCCTCCAAAAT | GGCTGTTGTCATACTTCTCATGG |

Table S2. Primary and Secondary Antibodies

| Type | Antibody | Application | Dilution | Species | Manufacture  And Catalog  Number |
| --- | --- | --- | --- | --- | --- |
| Primary | Anti-OCT4 | Immunofluorescence | 1:100 | mouse monoclonal | Santa Cruz  sc-5279 |
|  | Anti-SSEA4 | Immunofluorescence | 1:100 | Mouse  Monoclonal | Santa Cruz  sc-21704 |
|  | Anti-cTnT | Immunofluorescence; Flow cytometry | 1:100 | Mouse  Monoclonal | Abcam  Ab8295 |
|  | Anti-MYL2 | Immunofluorescence; Flow cytometry; | 1:100; 1:100; | Rabbit  Polyclonal | Proteintech  10906-1-AP |
|  | Anti-α-actinin | Immunofluorescence | 1:100 | Rabbit  Polyclonal | Abcam  Ab137346 |
|  | Anti-α-1-syntrophin | Western blot | 1:500 | Mouse  Monoclonal | Santa Cruz sc-166635 |
|  | Anti-Nav1.5 | Western blot | 1:500 | Rabbit  Polyclonal | AlomoneLabs ASC-013 |
|  | Anti-GAPDH | Western blot | 1:500 | Mouse  Monoclonal | Santa Cruz sc-365062 |
|  | Anti-ATP1A1 | Western blot | 1:500 | Rabbit polyclonal | Servicebio GB11400 |
| Secondary | Goat anti-Mouse IgG Alexa Fluor  594 | Immunofluorescence | 1:200 | Goat anti-  Mouse IgG | Invitrogen  A21145 |
|  | Goat anti-Rabbit IgG Alexa Fluor  488 | Immunofluorescence | 1:200 | Goat anti-  Rabbit IgG | Invitrogen  A32731 |
|  | Chicken anti-Rabbit IgG Alexa  Fluor 594 | Immunofluorescence | 1:200 | Chicken anti-  Rabbit IgG | Invitrogen  A21442 |
|  | Chicken anti-Mouse IgG Alexa  Fluor 488 | Immunofluorescence | 1:200 | Chicken anti-  Mouse IgG | Invitrogen  A21200 |

Table S3. The software used in this study

| Software | URL |
| --- | --- |
| GraphPad Prism 8.0 | <https://www.graphpad.com/features> |
| ImageJ_v1.8.0 | <https://imagej.net/ij/download.html> |
| Primer Premier 6.0 | <http://www.premierbiosoft.com/primerdesign/overview.html> |
| SnapGene 2.3.2 | <https://www.snapgene.com/> |
| FlowJo_V10.8.1 | <https://www.flowjo.com/flowjo/download> |
